# Community Paramedicine: 911 Alternative Destinations Are a Patient Safety Issue

**DOI:** 10.5811/westjem.2016.11.32758

**Published:** 2017-01-20

**Authors:** Nick T. Sawyer, John D. Coburn

**Affiliations:** * University of California, Davis, Department of Emergency Medicine, Sacramento, California; † The Permanente Medical Group, South Sacramento Kaiser, Department of Emergency Medicine, Sacramento, California; ‡ California American College of Emergency Physicians Board of Directors

The 2010 Patient Protection and Affordable Care Act (ACA) served as a conduit for many previously uninsured U.S. citizens to obtain health insurance; however, insurance does not necessarily equate to timely access to care. A 2015 study found that efforts by policymakers and health insurance plans to drive Medicaid patients out of emergency departments (ED) and into primary care clinics are not working. 1 More than half of all providers listed by Medicaid managed care plans could not offer timely appointments to enrollees, despite a provision in the ACA temporarily boosting pay to primary care physicians treating Medicaid patients. The median wait time was two weeks, but over one-quarter of providers had wait times greater than one month. Consequently, newly insured patients are increasingly seeking care in EDs and the reliance on emergency care remains stronger than ever. In a May 2015 poll, three-quarters of emergency physicians reported that emergency visits were going up. This represents a significant increase from just one year ago when less than half reported increases.^2^ Lastly, a recent analysis of health plans under the ACA revealed that one in five plans did not even list any emergency services on the list of covered benefits.^3^ This results in increased financial burden to patients when emergency care is provided by an “out-of-network” emergency physician, frequently leading to the patient receiving a “surprise” balance bill.

Increased demand for emergency services leads to longer wait times, crowding and increased patient boarding in the ED. All have been associated with several negative patient-oriented outcomes – from lower patient satisfaction scores to higher inpatient mortality rates.^4^ Recognizing this, multiple stakeholders are currently working to mitigate the ballooning crowding dilemma.

One approach gaining popularity is community paramedicine (CP). CP is a “model of community based health care in which paramedics function outside their customary emergency response and transport roles in ways that facilitate more appropriate use of emergency care resources and/or enhance access to primary care for medically underserved populations.”^5^ Interest in CP has substantially grown in recent years based on the belief that it may improve access and quality of care while also reducing costs.^5^

In February 2014, California’s Emergency Medical Services Authority (EMSA) submitted a proposal to the Office of Statewide Health and Planning (OSHPD) to train experienced paramedics and expand their scope of practice to include the ability to transport patients with specific conditions to alternative destinations (AD). Such destinations would include primary care, general medical clinics, urgent care centers, and other social or psychological services.

Proponents of CP maintain that such programs expand access to care in an era of primary care shortage, while improving quality and lowering healthcare costs. Further, they argue that utilizing paramedics in expanded roles is attractive because they are already trained to recognize and manage life-threatening conditions in out-of-hospital settings. This may facilitate more appropriate use of emergency care resources and/or enhance access to primary care. These claims require close scrutiny, however, as the effect of CP on ED utilization, cost savings and enhanced primary care access is still being assessed, and to date, limited data exist to support these claims.

CP is not a new idea. Programs have been piloted in several states including New Mexico, Nevada, Colorado, Texas, Maine and Pennsylvania. To quote Scot Phelps, a former paramedic and professor of disaster science, regarding a prior CP attempt in New Mexico, “We tried this in 1995 in Red River, New Mexico, and what we found, after spending hundreds of thousands of dollars, was that it didn’t actually save any money or improve any care. So [that community] abandoned it, and now coming eight years later it is the topic du jour.”^6^

Several concerns have been raised regarding CP, most notably, the risk of paramedic under-triage and transport of patients requiring emergency care to AD. AD projects involve previously unknown patients who may have one or more unknown illnesses, injuries, or psychosocial problems. Complex patients are common in the prehospital and ED setting. Standard paramedic practice focuses on recognition of patients’ unstable physiology and management with temporizing and lifesaving interventions until transport to an ED is complete. The ED is the controlled environment for complete stabilization, evaluation, diagnosis, and disposition with care coordination. The ED, contrary to most or all ADs, has extensive diagnostic and therapeutic resources to help ferret out the occult medical emergency.

## Under-Triage is a Patient Safety Issue

As reported in the *Annals of Emergency Medicine* in 2014, studies have revealed under-triage by paramedics when not transporting patients to AD.^7^ The potential for under-triage is real if there is a failure of a community paramedic to recognize a real emergency when it exists. Further, identifying non-emergent patients based on their initial presentation is hazardous. In a study by Raven et al, 11% of patients with “primary care treatable” visits required immediate intervention, 12.5% were admitted, and 3.4% went directly to the operating room emergently.^8^

According to Morganti et al., “Nearly all studies published to date have found significant rates of under-triage by EMS Personnel…” These investigators identified 13 research studies examining the ability of paramedics and EMTs to determine the need for transport to the ED. These studies reveal EMS AD under-triage rates from 3% to 32%. They commented that the ability of EMS professionals to safely determine nonemergency patient “has not been clearly established.” Included in these studies was one study describing a cohort of under-triaged patients, who EMS professionals felt did not require transport to the ED for care, and who subsequently required admission to the hospital (18%), including a subset who required admission to the intensive care unit (6%). These problems were attributed to EMS professionals misusing study guidelines, undertraining in proper use of the guidelines, and improper or unclear instructions within the guidelines that could result in under-triage. These studies also revealed poor agreement between EMS professionals and emergency physicians about who required transport to the ED for care. Additional training is not likely to eliminate the problem of under-triage.

## Alternative Destinations will Disproportionately Affect Critically Ill and Vulnerable Patient Populations

Patients who call 911 are more likely to be critically ill, elderly, and economically disadvantaged relying on public rather than private insurance.^9^ The patient population that arrives by ambulance does not reflect the general ED population. Whereas a proposed estimate of 13.7% of ambulance calls could be diverted to an urgent care center based on a Health Affairs study by Weinick et al., this study reviewed all ED visits rather than the population of patients who call 911.^10^ Rugar et al. analyzed ambulance transports and triage category and found less than 2% of patients arriving by ambulance had a triage category of less urgent or non urgent.^11^ Patients with a triage category of emergent were nine times more likely to arrive by ambulance, and with a triage category requiring immediate interventions, 50 times more likely to arrive by ambulance. This suggests a vast majority of ambulance transports are appropriate. The policy of diverting 911 patients away from EDs will not target low acuity visits. Studies suggest that it may target sick, vulnerable patients who already have limited access to care, and may further limit their access to specialty care. Even though EDs certainly have problems referring patients for specialty care, or achieving consultation during the ED visit, such referrals and consultations from ADs would most likely be even more difficult, if not impossible.

In conclusion, lowering healthcare costs for payers should not come at the expense of patient safety. Limiting access to high quality emergency and specialty care may show immediate cost savings to payers, but concerns remain over the longer term expense to patients and payers in terms of overall health outcomes. To date, the literature does not support paramedic-guided diversion of ambulance patients away from the ED to AD in terms of cost savings or equivalent health outcomes. As interest grows in CP programs, rigorous research methods should be applied to validate claims that CP is safe, improves quality and lowers healthcare costs.
